# Exploring the Impact of Blood Pressure Variability on Incident Atrial Fibrillation in Type 2 Diabetes∗

**DOI:** 10.1016/j.jacadv.2023.100405

**Published:** 2023-06-30

**Authors:** Eunice Yang, Olive Tang

**Affiliations:** a Arrhythmia Division, Inova Heart and Vascular Institute, Fairfax, Virginia, USA; b Division of Cardiology, Johns Hopkins Hospital, Baltimore, Maryland, USA; c General Internal Medicine, Johns Hopkins School of Medicine, Baltimore, Maryland, USA

**Keywords:** atrial fibrillation, blood pressure variability, diabetes, incident atrial fibrillation

**Figure:**
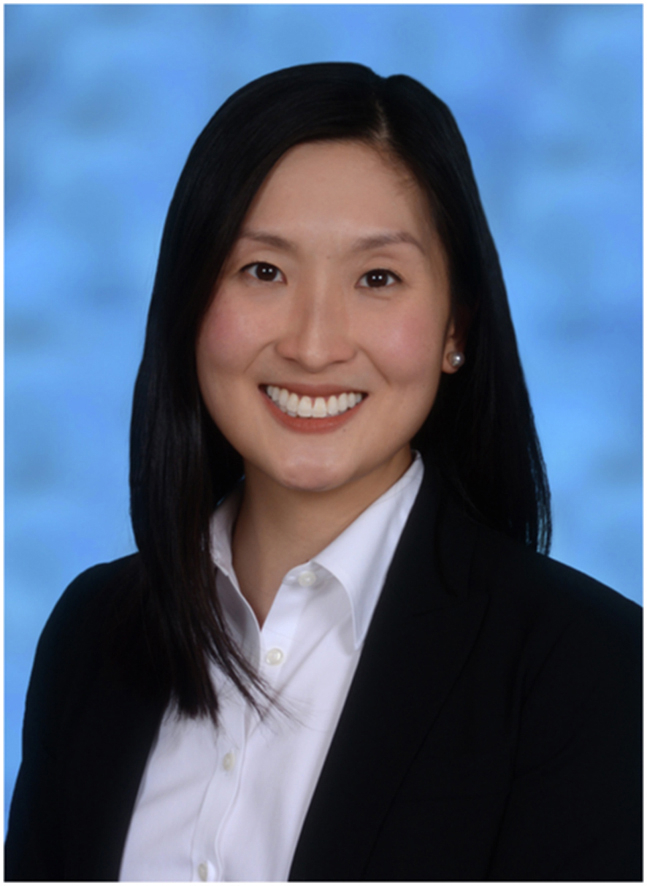

Atrial fibrillation (AF) affects an estimated 33 million individuals worldwide^1^ and significantly impairs quality of life. It is an independent risk factor for stroke, cardiovascular hospitalization, sudden death, and development of heart failure and dementia.^2^, ^3^, ^4^, ^5^ Therefore, elucidating risk factors associated with the development of AF provides an avenue for preventative health interventions.

Hypertension is a well-described risk factor for AF, and blood pressure screening is one of the most common measurements in clinical practice. On a beat-to-beat basis, blood pressure measurements in the same individual can vary markedly from −24 mmHg to +33 mmHg, owing to a multitude of intrinsic, extrinsic, and behavioral factors.^6^ Clinical studies investigating the impact of hypertension have traditionally sought to overcome the intrinsic variability in blood pressure measurements by using an averaged measurement with standardized measurement procedures.^7^^,^^8^ However, growing evidence suggests that the presence of increased blood pressure variability carries prognostic implications for cardiovascular and all-cause mortality outcomes, independent of blood pressure elevation alone.^9^, ^10^, ^11^, ^12^, ^13^ Previous research has suggested that increased blood pressure variability is associated with an increased risk for the development of AF in the general population,^14^ but these findings have yet to be corroborated in those with diabetes, who are known to be a higher cardiovascular risk population.

In this issue of *JACC: Advances*, Kaze et al^15^ investigate the link between increased blood pressure variability and incident AF in those with type 2 diabetes through a retrospective cohort analysis of the ACCORD (Action to Control Cardiovascular Risk in Diabetes) study—a multicenter 2-by-2 factorial randomized control trial investigating the impact of intensive blood pressure and/or glycemic control in patients with type 2 diabetes.^16^ Blood pressure variability was measured using at least 5 blood pressure measurements across multiple visits obtained between the 4th and 24th months of follow-up. Variability was defined as the intraindividual standard of deviation across visits, and also measured using coefficient of variation, and variability independent of the mean. They report that having higher blood pressure variability was associated with an increased incidence of AF during the follow-up period, defined as AF diagnoses using outpatient 12-lead electrocardiogram screening after month 24 of follow-up.

There are several strengths to highlight in the Kaze et al^15^ investigation of this association. There is inherent variability in blood pressure that can stem from methods in measurement. By leveraging the standardized blood pressure measurement protocol applied across all study participants in a randomized control trial, the investigators were able to minimize the measurement variations. They also importantly defined their exposure in a period of time prior to the onset of their follow-up period by leveraging the extensive cohort follow-up that has been ongoing in ACCORD. Through these careful considerations, Kaze et al^15^ have corroborated the findings previously shown in the general population.

Despite these strengths, there are also some limitations to the interpretation of these reported findings. The first limitation of this study arises from the selection of this study population, who have been randomized to a blood pressure control intervention as part of the original study design. This carries the intrinsic concern for confounding due to the antihypertensive treatments implemented through the study. It is not entirely clear from the manuscript whether only the glycemic randomization (which is reported in Table 1) or if, more importantly, the blood pressure randomization (not reported in Table 1) was controlled for in their analyses. One may wonder if those randomized to the aggressive blood pressure titration would experience more variability in the dosing of antihypertensive medications over time. Some of the antihypertensive medications used have shorter half-lives and the study design does not account for participant noncompliance or missed doses of the medications, which could contribute to observed variability over the course of these serial blood pressure measurements. By using serial blood pressure measurements obtained several months apart, there is the added variability based on the time of day of acquisition, missed doses of antihypertensives, as well as other tests conducted during scheduled follow-up. Furthermore, the antihypertensives used to achieve blood pressure reduction included beta-blockers: at the last follow-up visit, 61% of individuals in the intensive blood pressure treatment arm and 43% of individuals in the standard blood pressure treatment arm had been prescribed beta-blockers. Beta-blocker therapy has been reported to reduce to the onset of AF in select populations,^17^ which could significantly impact the diagnosis of incident AF using routine ECG screening, though Kaze et al^15^ attempt to address this confounder in their analysis through controlling for anti-arrhythmic use.

A second limitation arises in how Kaze et al^15^ adjust for clinical comorbidities. There were only 155 cases of incident AF after month 24 of follow-up.^15^ Comorbidities with strong associations with development of AF, such as heart failure and chronic kidney disease^3^^,^^18^^,^^19^ were not included until the final multivariable model, which may not have been appropriately powered for the number of covariates included. Given that ACCORD was not randomized to answer this particular question, the investigators astutely leveraged this as a cohort study. However, in that setting, the study investigators are no longer able to rely on randomization to control these significant confounding factors, which restricts our ability to infer associations between blood pressure variability and development of AF through the reported findings.

While these limitations exist, Kaze et al^15^ highlight an important gap in our mechanistic understanding of the association between higher blood pressure variability and stroke risk, which they posit may be mediated through the development of AF. Though their analysis does not address this question directly, the investigators have provided the stepping stones for further investigation looking at the possible impact of increased blood pressure variability as risk factor for thromboembolic events after accounting for AF, which is a well-known risk factor for stroke. Their findings raise the important question of whether blood pressure variability itself is a risk factor or simply a marker of underlying pathologic processes, including subclinical AF, which could certainly contribute to increased blood pressure variability due to beat-to-beat variability in diastolic filling time. Further investigation would be required to improve our understanding of this reported association.

Finally, an intrinsic limitation in the extrapolating findings regarding increased blood pressure variability lies in the translation to clinical practice, as there are currently no therapeutic interventions to target blood pressure variability independent of blood pressure control. Thus, elucidating the underlying pathology would be important to elucidate therapeutic targets for intervention. It is important to emphasize that there has been ample evidence that the control of hypertension is an important cardiovascular risk reduction strategy and patients with blood pressures above goal should not be maintained at their elevated blood pressure in efforts to minimize variability.

In sum, the analysis reported by Kaze et al^15^ corroborates and extends previously reported population-based findings linking increased blood pressure variability with the development of AF. Their findings raise important questions and highlight opportunities for future research, such as future investigations looking at the impact of blood pressure variability on thromboembolic stroke as an independent risk factor after adjustment for clinical and subclinical AF. While translating blood pressure variability to clinical practice or clinical interventions has consistently been challenging, the cumulative findings on the relationship between blood pressure variability and incident AF would suggest that individuals who develop an increase in blood pressure variability following otherwise stable blood pressure measurements may benefit from intensification of AF screening for earlier diagnosis and initiation of thromboembolic prophylaxis. As ambulatory rhythm and biometric monitoring becomes increasingly widespread, future studies in this space will be able to harness this technology to help refine our understanding of the association between blood pressure variability and development of atrial arrhythmias.

## Funding support and author disclosures

The authors have reported that they have no relationships relevant to the contents of this paper to disclose.
